# Optimization of Infrared Rotary Roasting Conditions for Immature Rice: Effects on Physicochemical and Cooking Qualities

**DOI:** 10.3390/foods15091578

**Published:** 2026-05-03

**Authors:** Lamul Wiset, Chainarong Chuayjum, Juckamas Laohavanich, Nattapol Poomsa-ad, David Julian McClements, Ekasit Onsaard, Wiriya Onsaard

**Affiliations:** 1 Innovative Agriculture Machinery and Post-Harvest Technology Research Cluster, Mahasarakham University, Maha Sarakham 44150, Thailand; lamul.w@msu.ac.th (L.W.); juckamas.l@msu.ac.th (J.L.); 2 Department of Food Technology, Faculty of Agriculture, Ubon Ratchathani University, Warin Chamrap, Ubon Ratchathani 34190, Thailand; chainarong.ch.57@ubu.ac.th (C.C.); ekasit.o@ubu.ac.th (E.O.); 3 Indigenous Food Research and Industrial Development Center, Ubon Ratchathani University, Ubon Ratchathani 34190, Thailand; 4 Drying Technology Research Unit, Faculty of Engineering, Mahasarakham University, Maha Sarakham 44150, Thailand; nattapol.p@msu.ac.th; 5 Biopolymers & Colloids Research Laboratory, Department of Food Science, University of Massachusetts, Amherst, MA 01003, USA; mcclemen@umass.edu

**Keywords:** immature rice, infrared roasting, response surface methodology, physicochemical properties, cooked-rice texture, sustainable processing

## Abstract

Immature rice is a distinctive cereal product widely consumed in Asian countries due to its natural green color, soft texture, unique flavor, and high nutritional value. However, its fragile structure and pigment sensitivity create significant processing challenges. This study investigates the effects of infrared (IR) roasting temperature (550–650 °C) and time (20–40 min) on the physicochemical, nutritional, and cooked-rice qualities of immature rice (*Oryza sativa* L., cv. RD6). A two-factor study with three level of factorials was designed and response surface methodology (RSM) was used to evaluate roasting variables and to identify optimal processing conditions (*p* ≤ 0.05). Increasing roasting severity decreased rice yield, moisture content, water activity, and chlorophyll content, while promoting grain darkening, increasing phenolic content, and enhancing cooked-rice expansion and hardness. Several responses exhibited significant linear and quadratic relationships with roasting conditions, with model coefficients of determination (R^2^) ranging from 0.676 to 0.829. Multi-response optimization using desirability analysis identified the optimal roasting condition as 650 °C for 20 min, which produced predicted values that closely matched experimental validation (*p* > 0.05). These results demonstrate that IR roasting provides an effective green-energy processing approach for producing value-added immature rice while maintaining desirable color, nutritional properties, and cooked-rice texture.

## 1. Introduction

Rice (*Oryza sativa* L.) is a major staple crop globally [1]. Rice grain ripening proceeds through several distinct physiological stages, including milk, soft dough, hard dough, and full maturity, which takes approximately 30–45 days after flowering [2,3]. During the soft dough stage, rapid accumulation of amyloplasts occurs in the endosperm, accompanied by starch deposition, dehydration, and active translocation of assimilates from leaves and culms [4]. At this intermediate developmental stage, rice grains remain physiologically immature but exhibit a unique combination of soft texture, green pigmentation, and elevated levels of bioactive compounds.

Numerous studies have reported that dough-stage or immature rice contains higher levels of protein, phenolic compounds, chlorophyll, β-carotene, tocopherols, γ-oryzanol, and γ-aminobutyric acid (GABA) than fully mature rice [3,5,6]. These bioactive compounds are associated with enhanced antioxidant activity and potential health benefits, including a reduced risk of cardiovascular disease, type 2 diabetes, and certain cancers [7]. Owing to these nutritional attributes, immature rice has long been processed into indigenous products such as “Khao Mao” in Thailand and similar products in Vietnam, Cambodia, and Lao PDR [3].

Despite its nutritional value, conventional processing of immature rice remains inefficient and difficult to control [8]. Traditional methods typically involve steaming followed by prolonged pan roasting over firewood, often exceeding 3 h per batch. This fuel-intensive process results in inconsistent product quality, excessive moisture loss, pigment degradation, reduced head rice yield, and substantial losses of heat-sensitive bioactive compounds [9,10]. In addition, reliance on biomass fuel raises environmental concerns and limits the scalability of immature rice processing in modern food systems.

Infrared (IR) roasting has emerged as a promising green-energy alternative for thermal food processing [11,12]. IR heating transfers energy directly to food materials via electromagnetic radiation, enabling rapid, uniform heating with reduced energy loss, shorter processing times, and improved controllability compared with conventional conduction-based roasting [11]. IR roasting has been successfully applied to peanuts, almonds, coffee, and cereal-based products, with improvements in product quality and energy efficiency reported [13,14]. However, systematic investigations on the application of IR roasting to immature rice remain scarce, and comprehensive optimization of processing conditions has not yet been reported. Because roasting intensity strongly affects grain integrity, color retention, nutritional quality, and cooked-rice texture, optimization of IR roasting parameters is essential.

Response surface methodology (RSM) is a powerful statistical approach for modeling and optimizing complex thermal processes involving multiple interacting variables and responses. RSM has been widely applied to optimize roasting conditions for coffee, cocoa, and oil-bearing seeds [15,16,17]. Nevertheless, its application to IR roasting of immature rice has not been explored. Therefore, the objective of this study was to investigate the effects of IR roasting temperature and roasting time on the physicochemical properties and cooked-rice quality of dough-stage glutinous rice (*Oryza sativa* L., cv. RD6). This study provides the first comprehensive optimization framework for IR roasting of immature rice and offers practical insights into sustainable, energy-efficient processing of value-added cereal products.

## 2. Materials and Methods

### 2.1. Raw Materials

Dough-stage glutinous paddy rice (*Oryza sativa* L., cultivar RD6) was harvested 15 days after flowering from a rice field located in Pho Sai District, Ubon Ratchathani province, Thailand. After harvesting, the immature paddy grains were cleaned to remove foreign materials and then stored at −18 °C until further use. All chemicals and reagents used in the analyses were of analytical grade.

### 2.2. Preparation of Immature Paddy (Dough Stage)

Frozen immature paddy was thawed at 4 °C for 24 h and equilibrated at room temperature (~25 °C) for 3 h. The grains were thoroughly washed with tap water to remove soil, husk residues, and impurities, after which immature and lightweight grains were removed. The cleaned paddy was steamed in a semi-automatic steamer for approximately 20 min (15 kg per batch). The steamed paddy was used for further experiments.

### 2.3. Infrared Roasting Process

**Figure 1:**
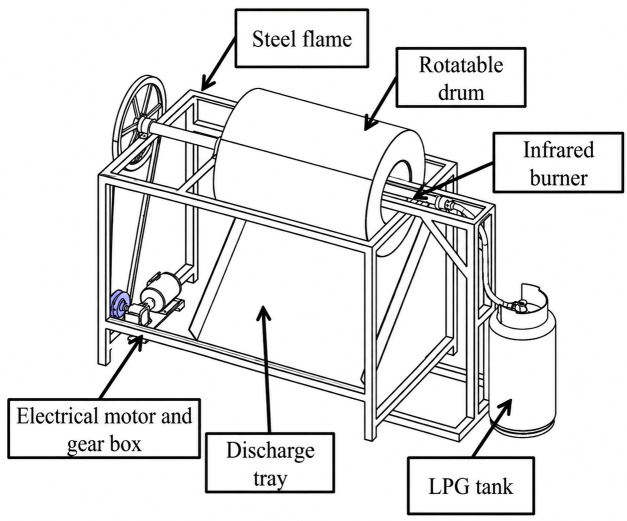
Rotary infrared drying machine used for immature rice roasting process, showing infrared burner, roasting drum, discharge tray, control unit, LPG tank, and drive motor [18].

Steamed immature paddy was roasted using a rotary infrared dryer (Figure 1) under conditions selected from a preliminary experiment. The IR roasting machine consisted of a stainless-steel rotating drum (0.6 m diameter × 1 m in length), a gas-fired type of IR burner (LPG gas), an electrical motor, and gear box for the rotating drum. The radiated electromagnetic waves in the length of far-infrared rays were 2.70–3.32 µm [18].

The roasting variables consisted of three roasting temperatures (550, 600, and 650 °C controlled by a gas burner and measured by type K thermocouples), and three roasting times (20, 30, and 40 min), with the drum rotating speed fixed at 60 rpm. A 3^2^ full factorial design was employed to evaluate the effects of IR roasting conditions, roasting time and temperature, and the interaction effect of two factors, on the physicochemical and cooked-rice quality of immature rice. Each roasting batch contained 2 kg of immature paddy, and experiments were conducted according to a full factorial design in random order with three replications. After roasting, the paddy samples were transferred to a sealed container and tempered for 30 min to allow moisture equilibration. The grains were then shade-dried until water activity (aw) was ≤0.65, corresponding to a moisture content ≤ 14% (wet basis). The dried roasted paddy was dehulled using a brown rice milling machine. Roasted immature rice samples were packed in aluminum foil pouches and stored at room temperature for physical quality analysis, while samples for chemical analysis were stored at −18 °C.

### 2.4. Physical Quality Measurements

#### 2.4.1. Rice Yield and Head Rice Yield

Rice yield was calculated from the ratio of the dry weight of immature rice obtained after roasting to the initial weight of raw immature paddy. Head rice yield was determined as the proportion of impact grains that remained unbroken after milling. The percentages of rice yield and head rice yield were calculated using the following equations:

(1) Rice yield (%) = [(Weight of dry immature rice)/(Weight of raw immature paddy)] × 100

(2) Head rice yield (%) = [Weight of milled rice/Total weight of milled rice] × 100

All measurements were performed in triplicate, and results are expressed as mean values.

#### 2.4.2. Color

Color attributes (L*, a*, b*) of roasted immature rice were measured using a HunterLab ColorFlex Model 45/0 colorimeter (HunterLab, Reston, VA, USA). Measurements were performed in the CIE Lab* color space, where: L* represents lightness (0 = black, 100 = white), a* represents the red–green axis (+a = red, −a = green), and b* represents the yellow–blue axis (+b = yellow, −b = blue). The instrument was calibrated with a standard white calibration tile (L = 92.91, a* = −1.20, b* = −0.08) using D65 illuminant (Color Global Co., Ltd., Bangkok, Thailand). Roasted rice samples were placed in a quartz cell, and five random readings were recorded for each sample. The mean value was used to ensure measurement accuracy.

### 2.5. Chemical Quality Measurements

#### 2.5.1. Moisture Content and Water Activity

Moisture content was determined gravimetrically according to a standard method (AOAC, 1990) [19]. Approximately 3 g of sample was dried at 105 °C in a hot-air oven (Memmert, UN10, Schwabach, Germany) until a constant weight was achieved. Moisture content was calculated on a wet basis. All measurements were performed in triplicate, and results are expressed as mean values. Water activity was measured using a water activity meter (Novasina LabMaster-aw neo 21CFR11, Lachen, Switzerland). Each sample was analyzed in triplicate, and the mean a_w_ value was reported.

#### 2.5.2. Chlorophyll Content

Chlorophyll content was quantified following a standard method (AOAC, 1990) [19]. Sample extracts were analyzed using a UV–visible spectrophotometer (T60 UV/Visible, PG Instruments Ltd., Houston, TX, USA) by measuring absorbance at 660 nm and 642.5 nm. Total chlorophyll content was calculated and expressed as mg/100 g (dry weight basis). All determinations were conducted in triplicate.

#### 2.5.3. Phenolic Compound Content

Total phenolic compounds were determined using the Folin–Ciocalteu method described by Slinkard and Singleton et al. (1977) [20]. Absorbance was measured using a UV–visible spectrophotometer, and the results are expressed as mg gallic acid equivalent (GAE) per 100 g (dry weight basis). Analyses were performed in triplicate, and the results are presented as mean values.

### 2.6. Cooked Immature Rice Quality Analysis

#### 2.6.1. Preparation of Cooked Immature Rice

Roasted immature rice was cleaned to remove foreign materials. The dried grains were weighed and mixed with water at a ratio of 1:1 (*w*/*w*). The mixture was transferred into aluminum cups and cooked by steaming over a gas stove for 20 min. After cooking, the samples were placed in plastic containers and allowed to equilibrate at room temperature prior to subsequent physical quality analysis.

#### 2.6.2. Bulk Density

Bulk density was determined using a 2 g portion of immature rice, which was transferred into a 50 mL graduated cylinder. The occupied volume was recorded without tapping to avoid compaction. Bulk density (ρ) was calculated as the mass of the sample (g) divided by the measure volume (mL). All measurements were performed in triplicate, and the results are expressed as mean values (g/mL).

#### 2.6.3. Expansion Index

The expansion index of cooked immature rice was determined following the method of Juliano (1985) [21] with slightly modification. Approximately 2 g of immature rice was placed in a graduated glass tube, and the initial height (raw rice height) was recorded. Ten milliliters of distilled water were then added, and the sample was soaked at room temperature (~25 °C) for 30 min. The tube was then heated in a water bath at 100 °C for 12 min. After cooking, the final height of the expanded rice was measured. The expansion index was calculated using the following equation:

(3) Expansion index = [(Height of cooked rice)/(Height of raw rice)]

Measurements were performed in triplicate, and the results are expressed as mean values.

#### 2.6.4. Texture Profile Analysis

The textural properties of cooked immature rice were determined using a Texture Analyzer (TA.XT Plus, Stable Micro Systems, Godalming, UK) following the method of Yu et al. (2012) [22], with slight modifications. The instrument was equipped with a 50 N load cell and a square stainless-steel probe (7 × 7 cm). Cooked rice samples were placed in a square aluminum container (8 × 8 cm) designed to hold the samples during testing. Texture measurements were performed at a probe speed of 5 mm s^−1^ using a double-compression test mode to simulate mastication. The force–time profiles obtained from these compression tests were used to determine the following texture parameters: hardness (N), chewiness (kgf.mm), cohesiveness, and adhesiveness (kgf.mm).

### 2.7. Response Surface Methodology (RSM)

A 3^2^ full factorial design was employed to evaluate the effects of IR roasting temperature and time on the physical, chemical, and cooked-rice qualities of immature rice. Analysis of variance (ANOVA) was performed for each response variable to assess the significance of roasting temperature and time based on F-statistics at a probability level of *p* ≤ 0.05. The independent variables were IR roasting temperature (x_1_: 550, 600, and 650 °C) and roasting time (x_2_: 20, 30, and 40 min). In addition, the use of three levels of each factor with equal spacing allowed the detection of curvature in responses, which was important for modeling quality changes during roasting, and can be fitted using polynomial model [23]. The dependent variables (responses, $y_{n}$) included the physical, chemical, and cooked-rice quality attributes of immature rice. Multiple regression analysis was conducted using MINITAB^®^ Release 16.1 (Minitab Inc., State College, PA, USA) to fit a second-order polynomial model incorporating linear, quadratic (second-order polynomial), and interaction terms as shown in Equation (4) [17]. Regression coefficients (β) were estimated for each response, and model adequacy was assessed using lack-of-fit tests and the coefficient of determination (R^2^). Only statistically significant terms were retained for the interpretation of response surfaces and contour plots.

(4) $$y_{n}=\beta_{0}+\beta_{1}x_{1}+\beta_{2}x_{2}+\beta_{11}x_{1}^{2}+\beta_{22}x_{2}^{2}+\beta_{12}x_{1}x_{2}$$

where β_0_ is the intercept; β_1_ and β_2_ are the linear coefficients; β_11_ and β_22_ are the quadratic coefficients; and β_12_ is the interaction coefficient.

### 2.8. Optimization and Validation of Optimum Infrared Roasting Conditions

Optimization of IR roasting conditions was conducted using superimposed contour plots generated from the fitted second-order polynomial models. Selected quality attributes were employed as optimization criteria because they collectively represent grain integrity, color preservation, nutritional value, and cooked-rice texture. For each response, acceptable upper and/or lower limits were defined based on quality requirements of commercial immature rice, relevant literature, and experimental observations. Contour plots were generated over the experimental ranges of infrared roasting temperature (550–650 °C) and roasting time (20–40 min), and superimposed to identify the common feasible region that simultaneously satisfied all predefined criteria. The optimum IR roasting condition was determined at the intersection of these acceptable regions. Model validation was performed by conducting additional roasting experiments at the predicted optimal condition. Predicted response values were compared using *t*-tests and non-significant differences (*p* > 0.05) were considered evidence of adequate model predictability. In addition, residual diagnostic plots, including (i) plots of residuals versus predicted values, and (ii) observed versus predicted response plots, were examined to further assess model adequacy.

### 2.9. Activity-Based Energy Consumption Analysis

An activity-based costing (ABC) approach was applied to compare energy use for optimal immature rice roasting conditions [24]. In this study, the roasting activity, i.e., processing time, is the principle activity driver for ABC methodology. The roasting process was assessed as energy consumption, fuel use and carbon emission to support process-level efficiency evaluation [25]. The consumption analysis formula are as follows:

(5) EC=P×t

where EC is energy consumption per batch (kWh), P is burner heat output (kW), and t is roasting time (h).

(6) FU=FC×t

where FU is fuel use per batch and FC is fuel consumption rate.

(7) CE=FU×EF

where CE is carbon emission per batch and EF is the fuel-specific emission factor (0.3 kg/kWh).

## 3. Results and Discussion

### 3.1. Physical and Chemical Quality of Infrared-Roasted Immature Rice

#### 3.1.1. Appearance

**Figure 2:**
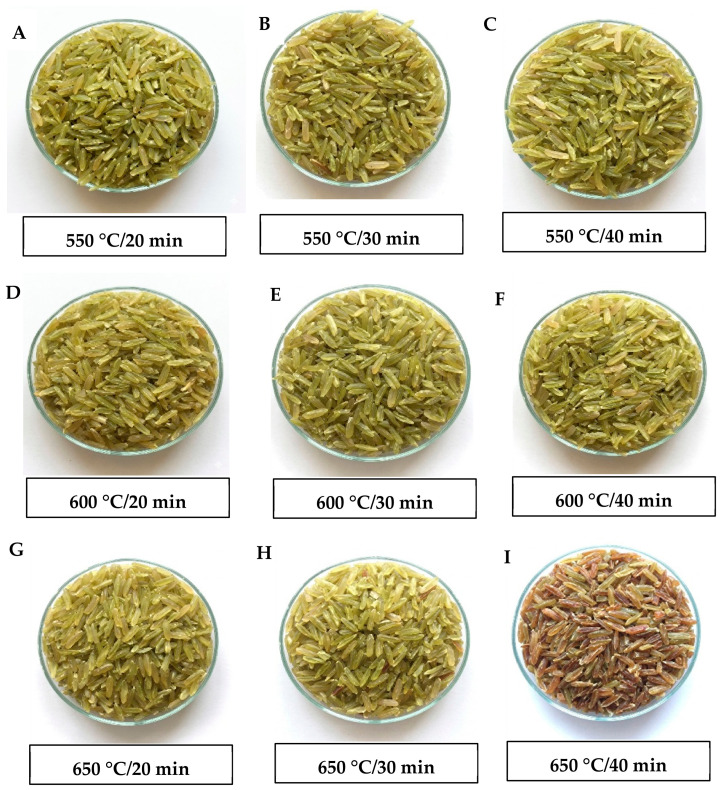
Visual appearance of immature rice roasted under different infrared temperatures and roasting times. (**A**) 550 °C—20 min, (**B**) 550°C—30 min, (**C**) 550°C—40 min, (**D**) 600°C—20 min, (**E**) 600°C—30 min, (**F**) 600°C—40 min, (**G**) 650°C—20 min, (**H**) 650°C—30 min, (**I**) 650°C—40 min.

The appearance of roasted immature rice samples prepared using different IR roasting conditions is shown in Figure 2. Clear differences in surface color and grain morphology were observed as roasting temperature and time increased. At lower roasting intensities (550 °C for 20–40 min), the grains retained their greenish color and exhibited smooth, intact surfaces with minimal visual structural changes (Figure 2A–C). In contrast, higher roasting temperatures (600–650 °C) combined with longer roasting times (up to 40 min) progressively produced a darker brown coloration, reflecting increased Maillard browning and pigment degradation (Figure 2D–I).

#### 3.1.2. Physical Quality

A 3 × 3 full factorial experimental design was employed to determine the optimal IR roasting conditions for immature green rice, using two independent variables: IR roasting temperature (500, 600, 650 °C) and roasting time (20, 30, 40 min). These variable levels were selected based on preliminary trials, generating nine treatment combinations to comprehensively evaluate the interaction effect of roasting variables on the physical quality of infrared-roasted rice. ANOVA using SPSS software (Version 15.0, SPSS Inc., Chicago, IL, USA) revealed significant main and interaction effects (*p* ≤ 0.05) on rice yield, head rice yield, and color values (L*, a*, b*).

As shown in Table 1, rice yield (%) significantly decreased with increasing roasting temperature and time (*p* ≤ 0.05), which can be attributed to intensified moisture loss, kernel fissuring, and starch gelatinization. A similar declining tread was observed for head rice yield (%), which was significantly influenced by both roasting factors and their interaction (*p* ≤ 0.05), reflecting heat-induced structural weakening of rice endosperm. Increased brittleness enhanced susceptibility to fracture during dehulling, thereby reducing milling efficiency and head rice recovery. These results indicate that excessive IR roasting adversely affects processing yield, whereas moderate roasting conditions are more suitable for maintaining grain integrity and mass retention of immature rice.

Color analysis showed that L* values declined significantly with roasting time and interaction effects (*p* ≤ 0.05), indicating a progressive darkening due to intensified non-enzymatic browning reactions. Roasting temperatures alone did not significantly influence L*.

In contrast, a* values increased significantly across all factors (*p* ≤ 0.05), reflecting the development of reddish-brown pigments associated with Maillard reactions and chlorophyll degradation under high thermal stress. The b* value increased significantly only with roasting time (*p* ≤ 0.05), consistent with prolonged exposure to the browning reaction. However, the relatively narrow variation in b* (26.0–27.7), representing the yellow hue of immature rice, suggests partial retention of pigment characteristics despite pheophytin formation from chlorophyll degradation during IR roasting. Overall, these color changes are consistent with the visual observation in Figure 2 and confirm that excessive IR roasting led to undesirable darkening, whereas moderate roasting conditions better preserve the natural color of immature rice.

#### 3.1.3. Chemical Quality

The chemical quality of infrared-roasted immature rice was evaluated using a 3^2^ full factorial design, focusing on moisture content (%), water activity, chlorophyll content, and total phenolic compounds (Table 1). Analysis of variance (ANOVA) revealed significant main effects of roasting temperature and time, as well as their interaction (*p* ≤ 0.05), on moisture content, water activity, and chlorophyll content, all of which decreased with increasing roasting severity. The substantial moisture loss observed under severe IR roasting conditions can be attributed to intensified radiative heat transfer, enhanced surface evaporation, and accelerated internal moisture diffusion driven by steep thermal gradients. The progressive decline in a_w_ reflects a simultaneous reduction in free and bound water, thereby improving resistance to microbial growth and enhances product safety and shelf stability.

These findings are consistent with previous studies on thermally roasted cereal and seed products. For instance, coffee beans exhibited progressive moisture reduction with increasing roasting temperature and time [26], and both moisture content and water activity have been reported to decline during thermal roasting [27]. Microwave roasting has likewise been shown to promote rapid moisture removal from oilseeds [28]. In general, roasting is characterized by simultaneous heat and mass transfer processes that promote vapor migration and moisture loss [29,30].

The progressive decline in chlorophyll content with increasing roasting severity is consistent with previous studies showing that thermal treatment promotes chlorophyll degradation through replacement of the central Mg^2+^ ion in chlorophyll by H^+^, resulting in the formation of pheophytin as well as other degradation products, such as pyropheophytin and related derivatives. These reactions are influenced by processing conditions, particularly temperature, pH, and moisture, and are commonly associated with a color shift from bright green to olive-brown [31].

Conversely, total phenolic content exhibited a complex response to roasting conditions. A significant decrease (*p* ≤ 0.05) was observed with increasing roasting temperature and time, except at the highest temperature (650 °C), where an apparent increase in TPC was detected. The initial decline under mild-to-moderate roasting conditions is likely attributable to the thermal degradation and oxidation of native polyphenols, as reported for thermally processed cereals and seeds [32]. However, the increase in TPC observed under severe roasting conditions should be interpreted with caution. The Folin–Ciocalteu assay is non-specific and can react with a wide range of reducing compounds, including Maillard reaction products (MRPs) formed during thermal processing. Therefore, the observed increase in TPC may partly reflect the formation of Folin-reactive non-phenolic compounds, rather than a true increase in phenolic content. Additionally, thermal processing may enhance the release of bound phenolics from the plant cell matrix, which could contribute to the measured values [6,33]. Similar dual behavior, partial degradation of native phenolics combined with extractability and MRP formation, has been reported for roasted sesame seeds, coffee, and other grain-based products [32,34]. Accordingly, the TPC value reported in this study represents the apparent phenolic content, and roasting conditions should be optimized to balance color retention, antioxidant potential, and overall product quality.

### 3.2. Cooked-Rice Quality of Infrared-Roasted Immature Rice

The textural and physical properties of cooked immature rice obtained after roasting at different temperatures and times were evaluated in terms of expansion index, bulk density, and texture profile (hardness, chewiness, cohesiveness and adhesiveness). Analysis of variance (ANOVA) confirmed significant main and interaction effects of roasting temperature and time on bulk density and the expansion index (*p* ≤ 0.05) (Table 2).

The observed increase in bulk density with increasing roasting severity may be related to changes in the rice grain structure and packing behavior. Bulk density depends not only on the intrinsic density of individual grains but also on their ability to pack efficiently within a given volume. Although thermal treatment may increase internal porosity at the microstructural level, the simultaneous effects of moisture loss, kernel shrinkage, and reduced surface stickiness may enhance packing efficiency and decrease inter-particle void space, resulting in a higher bulk density [35].

Across all treatments, the expansion index ranged from 1.9 to 2.3 and increased with both roasting temperature and roasting time. At 550 °C, the expansion index increased slightly from 1.9 to 2.0, whereas at 600 °C the increase was more pronounced from 2.0 to 2.3. The highest expansion was observed at 650 °C, rising from 1.9 to 2.3, which is comparable to values reported for parboiled rice [36].

The increase in the expansion index at higher roasting intensities may be associated with heat-induced modifications in starch structure that promote water absorption and swelling during cooking. Previous studies have suggested that thermal treatment can alter starch organization and reduce crystalline order, thereby enhancing hydration and expansion behavior [37]. However, because no direct structural analyses of starch (e.g., DSC, XRD, or SEM) were performed in this study, this explanation should be considered tentative and interpreted as a plausible mechanism based on the literature rather than direct experimental evidence.

These results indicate that higher IR temperatures and longer roasting times increase both bulk density and the expansion index of cooked immature rice. However, these conditions must be balanced with other quality attributes (yield, color, texture) to avoid over-roasting.

Moreover, the effects of IR roasting temperature and roasting time on the textural properties of cooked immature rice are also shown in Table 2. Hardness increased significantly with both factors independently (*p* ≤ 0.05), whereas their interaction was not significant. This increase is attributed to severe IR roasting promoting protein denaturation and protein–starch matrix densification, reduced amylose leaching, and moisture redistribution, resulting in a denser and more rigid grain matrix after cooking. Chewiness increased significantly with roasting time and interaction effects (*p* ≤ 0.05), but roasting temperature did not affect chewiness (*p* > 0.05). It was observed that chewiness increased sharply with roasting at 650 °C, reaching a maximum value of 15.9 kgf.mm at 40 min. This behavior can be attributed to the combined effects of increased hardness and structural rigidity, which elevate the energy required to deform and masticate cooked rice. Cohesiveness of cooked infrared-roasted rice showed a significant time-dependent elevation (*p* ≤ 0.05), but roasting temperature and interaction effects proved non-significant (*p* > 0.05), indicating that roasting intensity alone was insufficient to alter this attribute within the tested range. Roasting time at 40 min across all temperatures (550, 600, 650 °C) yielded markedly higher values than 20 and 30 min (*p* ≤ 0.05). This enhancement reflects prolonged heat-induced starch recrystallization and amylopectin chain realignment, which strengthen the intra-granule gel network during cooking.

Comparable increases in hardness and chewiness following high-temperature pretreatments have been widely reported for infrared-, hot-air-, and microwave-processed rice and cereal grains, where heat-induced starch gelatinization, protein denaturation, and moisture migration collectively influence the cooked texture, leading to a firmer and more resilient structure [22,35,38]. Similar mechanisms of starch reassociation leading to increased cohesiveness, hardness, and chewiness have also been reported for thermally processed rice and cereal starch systems [39].

In contrast, adhesiveness remained invariant across all roasting conditions (temperature, time, interaction, *p* > 0.05). This stability suggests that the roasting condition preserved the characteristic surface tackiness of cooked rice regardless of processing severity. Adhesiveness is primarily governed by complex interactions among surface starch leaching, moisture availability, and matrix rigidity rather than roasting intensity [40,41]. This minimal modification to outer amylose exudate layers and bran-surface hydrophilicity is critical for maintaining the traditional sticky-rice mouthfeel in culinary applications like desserts or snacks.

Overall, the experimental data demonstrate that hardness and chewiness are the most sensitive texture attributes to IR roasting conditions, whereas cohesiveness and adhesiveness show comparatively minor responses. High roasting temperatures combined with long roasting times significantly increased firmness and chewing resistance, which may negatively affect consumer acceptability if excessive. Therefore, moderate IR roasting conditions are preferable for achieving a balanced cooked-rice texture, while avoiding excessive hardness and chewiness.

### 3.3. Model Fitting and Analysis of Variance for Response Surface Plots

The effects of infrared (IR) roasting temperature and time on the physical, chemical, and cooking quality attributes of roasted and cooked immature rice were evaluated using multiple regression analysis. Model adequacy was assessed by analysis of variance (ANOVA), coefficients of determination (R^2^), lack-of-fit tests, and the significance of individual regression coefficients (Table 3). Based on these criteria, rice yield, head rice yield, a* value, chlorophyll content, total phenolic content (TPC), expansion index, hardness, cohesiveness, and adhesiveness were initially considered for model fitting.

Several attributes—including L*, b*, moisture content, water activity, bulk density, and chewiness—although statistically significant in ANOVA, exhibited significant lack-of-fit (*p* ≤ 0.05), low R^2^ values, and weak regression coefficients. Nevertheless, the lack-of-fit for roasted rice yield was not significant (*p* > 0.05), suggesting no evidence of serious model inadequacy, but the model showed a relatively low coefficient of determination (R^2^ = 0.487), indicating a limited predictive strength for this response. These responses were therefore excluded from further modeling, indicating that second-order polynomial equations were insufficient to describe their variability under the studied roasting conditions.

Head rice yield, a* value, chlorophyll content, and total phenolic content were significantly affected by both linear and quadratic terms of roasting temperature and time (*p* ≤ 0.05), indicating nonlinear responses to IR roasting severity. These models showed moderate-to-high R^2^ values (0.68–0.830). However, a significant lack-of-fit (*p* ≤ 0.05) was observed, suggesting that localized deviations—particularly at extreme roasting conditions—were not fully captured by the second-order models. Regression coefficients revealed that temperature exerted a stronger influence on head rice yield and TPC, whereas roasting time dominated changes in a* value and chlorophyll degradation, consistent with pigment transformation kinetics and cumulative thermal exposure.

For cooked rice attributes, lack-of-fit tests were not significant (*p* > 0.05) for the selected responses. The expansion index and hardness exhibited high R^2^ values (0.83 and 0.75, respectively) and statistically significant regression coefficients, confirming robust model performance. The expansion index was influenced by both linear and quadratic terms of temperature and time, with time showing the greater effect magnitude. In contrast, hardness was primarily governed by linear effects, with roasting temperature contributing more strongly than time. Quadratic terms were not significant for hardness, indicating limited curvature in the response surface. Although the lack-of-fit for adhesiveness and cohesiveness was not significant (*p* > 0.05), these responses showed low R^2^ values (0.23 and 0.046) exhibited limited predictive reliability, meanwhile adhesiveness showed no meaningful correlation with roasting variables. Therefore, the expansion index and hardness were selected as predictive responses, while adhesiveness and cohesiveness were excluded from response surface plotting and optimization.

**Figure 3:**
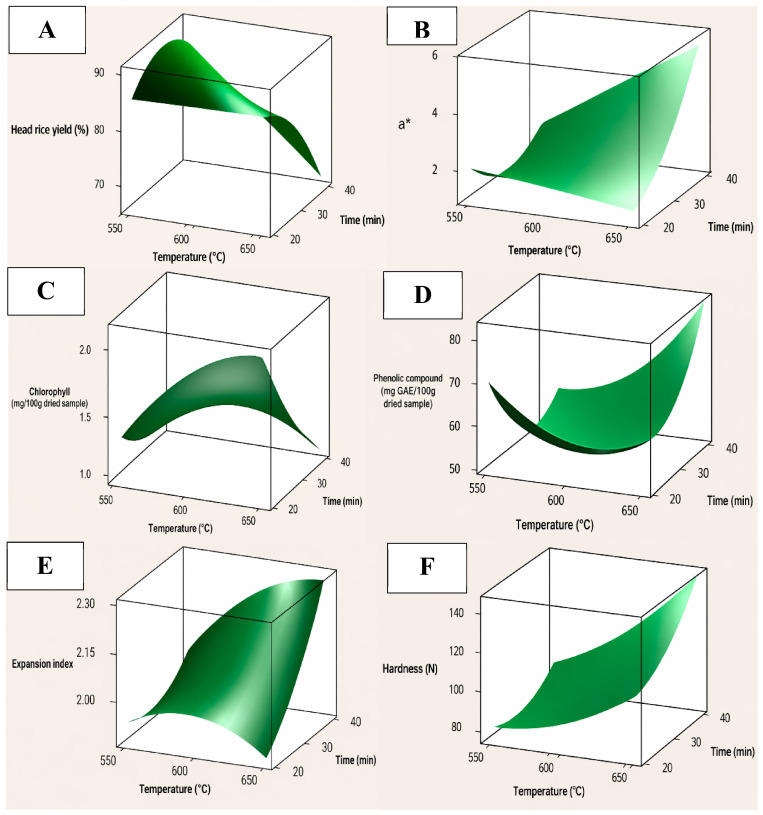
Three-dimensional response surface plots showing the effects of infrared roasting temperature (°C) and roasting time (min) on roasted immature rice and cooked-rice properties: (**A**) head rice yield, (**B**) a*, (**C**) chlorophyll content, (**D**) total phenolic content, (**E**) Expansion index, (**F**) hardness.

Response surface contour plots (Figure 3) illustrate the combined effects of roasting temperature and time on selected quality attributes. Head rice yield declined markedly with increasing roasting severity, particularly under prolonged exposure at high temperatures, reflecting thermally induced structural weakening and fissure development (Figure 3A). The a* value increased progressively (Figure 3B), indicating a shift toward reddish-brown tones driven by pigment degradation and non-enzymatic browning reactions, with roasting time exerting a dominant influence at intermediate conditions.

Chlorophyll content decreased consistently with increasing roasting intensity, displaying a smooth downward curvature that reflects cumulative thermal degradation to pheophytins and related derivatives (Figure 3C). Despite moderate R^2^ values and minor deviations at extreme conditions, the response surface demonstrated coherent trends within the practical processing domain.

Similarly, TPC exhibited a nonlinear response (Figure 3D), suggesting the coexistence of two completing thermal mechanisms. The moderate roasting enhanced phenolic availability by cell-wall softening, matrix disruption, and partial hydrolysis of structural polysaccharides, which can release bound phenolics. Similar heat-induced increases in measurable bound phenolics have been reported in cereal systems after cooking, indicating that thermal treatment can enhance phenolic accessibility before degradation becomes dominant [42]. Meanwhile, roasting promotes the Maillard reaction, where the reaction rate is governed by temperature, time, water activity, and matric composition; the resulting Maillard reaction products include melanoidins and related product compounds. Thus, these reaction products may contribute to higher Folin–Ciocalteu values and antioxidant responses [43]. However, under more severe roasting conditions, the balance shifts toward oxidation, thermal cleavage, polymerization, and interactions of phenolics with proteins and sugars, which reduce the measurable pool of native phenolics resulting in a concave response. This mechanistic interpretation reinforces that IR roasting condition should be optimized for balancing roasting intensity to maximize nutritional value.

For cooked rice, the expansion index increased with roasting severity, particularly at longer roasting times and higher temperatures (Figure 3E), indicating enhanced starch disruption and water absorption capacity during cooking. Hardness increased sharply under severe roasting conditions (Figure 3F), reflecting starch retrogradation, protein denaturation, and moisture redistribution. A significant interaction between temperature and time confirmed that excessive roasting combinations rapidly intensified textural firmness beyond desirable levels.

Overall, the response surfaces demonstrate that roasting temperature and time act synergistically rather than independently, governing both quality enhancement and degradation mechanisms. The second-order polynomial models effectively captured the dominant trends—declining head rice yield and chlorophyll, increasing a*, expansion index, and hardness, and the nonlinear behavior of phenolic compounds—within the operational range. Although localized lack-of-fit occurred for certain attributes at extreme conditions, the models remain statistically and technologically reliable within the optimization window.

Accordingly, the inclusion of chlorophyll content and total phenolic compounds alongside head rice yield, a* value, expansion index, and hardness in the desirability-based optimization is both statistically justified and functionally relevant. This integrated selection captures structural integrity, visual quality, nutritional functionality, and cooking performance, thereby enhancing the robustness, interpretability, and industrial applicability of the optimized IR roasting conditions for immature rice. Nevertheless, sensory analysis of immature roasted rice is also an important component for product optimization, as it links physicochemical and textural quality attributes with consumer acceptance and commercial feasibility. Therefore, future work should include ethically approved sensory evaluations to confirm the consumer acceptability and practical market relevance of the optimized product.

### 3.4. Optimization of Infrared Roasting Conditions for Immature Rice

IR roasting conditions for immature rice were optimized using response surface methodology (RSM) in combination with superimposed contour plots derived from second-order regression models (Table 4). The selected responses, head rice yield, a* value, chlorophyll content, total phenolic compounds, expansion index, and hardness, collectively represent critical indicators of grain structural integrity, appearance, nutritional value, and cooked-rice quality. The fitted models demonstrated predictive ability, with coefficient of determination (R^2^) values between 0.68 and 0.83, confirming their suitability for multi-response optimization. To define desirable IR roasting conditions, response limits were established using a combination of literature-supported quality relevance, practical product requirements, and experimentally observed response domains (Table 4). Head rice yield was constrained to a value above 60% because grain integrity is a major determinant of milling quality and economic value, and head rice yield is widely recognized as a standard indicator of commercial rice quality. In addition, broken kernels are substantially less valuable than head rice, supporting the use of a minimum threshold for acceptable structural retention [25]. The limits for the a* value and chlorophyll content were selected to preserve the characteristic green appearance of immature rice [3], since chlorophyll degradation during thermal processing is directly associated with color loss and browning [44]. From a nutritional perspective, total phenolic compounds were constrained within an acceptable range to preserve the functional value of immature rice, as developing rice grains are known to contain substantial levels of phenolic bioactive compounds [6], where excessive roasting may promote thermal degradation. For the expansion index and hardness, the optimization criteria were defined by comparison with the cooked-rice characteristics of immature rice prepared using the traditional firewood-roasting method. Because this conventional process represents the typical product quality accepted in practice, the selected ranges were intended to maintain the textural properties within a practically relevant range.

**Figure 4:**
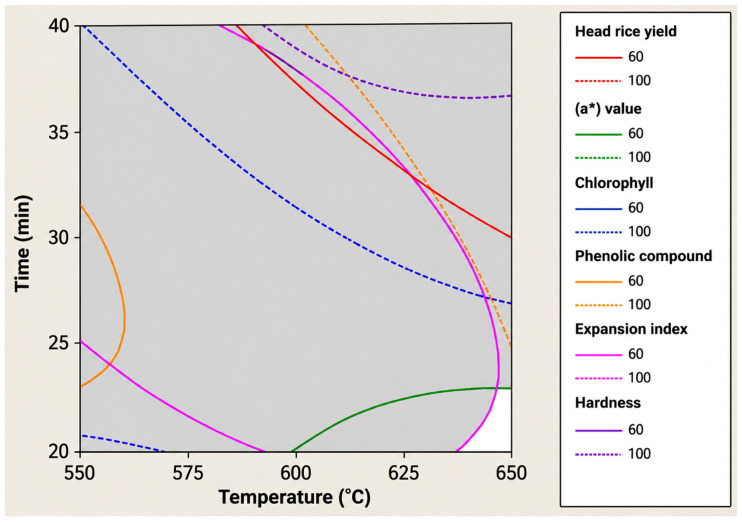
Superimposed contour plots were used to determine the optimum infrared roasting conditions of immature rice based on simultaneous optimization of head rice yield, a* value, chlorophyll content, total phenolic compounds, expansion index, and hardness.

Superimposition of the contour plots for all response variables (Figure 4) revealed a distinct feasible region in which all predefined quality criteria were simultaneously satisfied. This feasible region highlights the intrinsic trade-off involved in IR roasting, as improvements in certain attributes (e.g., expansion index or phenolic content) may coincide with deterioration in others (e.g., head rice yield or color). Using the Minitab Overlay Plot function, the optimal roasting condition was identified at an IR temperature of 650 °C and a roasting time of 20 min, where all response variables fell within their respective target ranges.

### 3.5. Model Validation of Optimum Roasting Conditions

The validity of the optimum roasting conditions identified by RSM and the superimposed contour plots was evaluated by comparing predicted response values with those obtained from experimental trials conducted under the optimal conditions (650 °C for 20 min). The predicted and experimentally measured values of key quality attributes are summarized in Table 5. Overall, no significant differences were observed between the predicted and experimental values for all response variables, indicating the reliability and predictive accuracy of the developed regression models.

**Figure 5:**
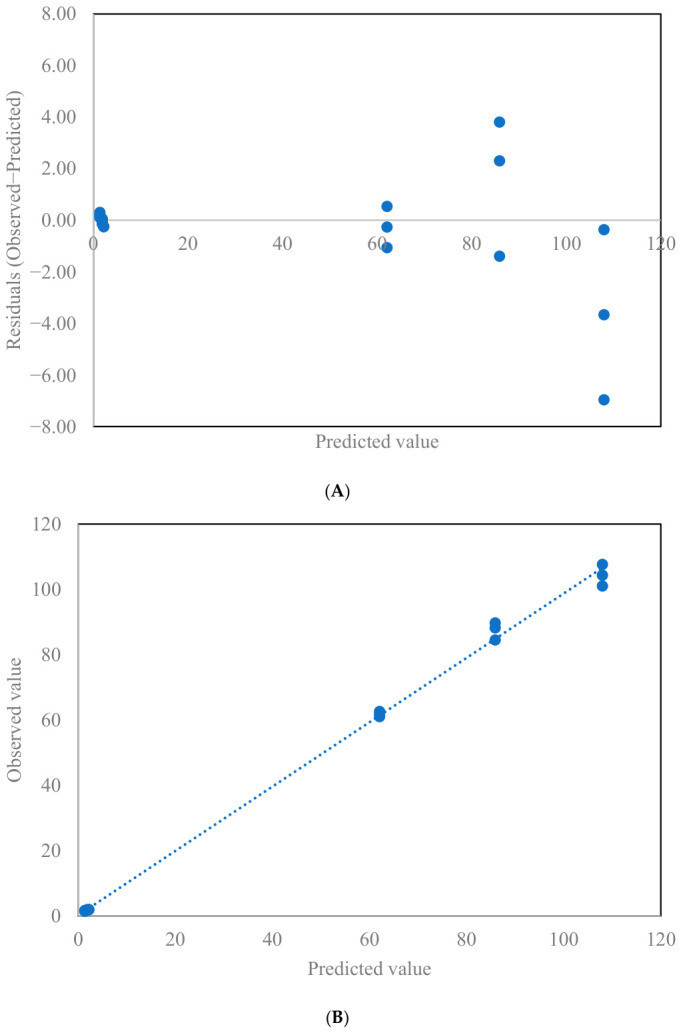
The residual diagnostic plots: (**A**) plots of residuals versus predicted values, and (**B**) observed versus predicted responses for validation of the optimized immature rice roasting conditions.

The experimentally determined head rice yield (88.2%) was slightly higher than the predicted value (85.9%), confirming effective preservation of grain integrity under the optimized roasting condition. Similarly, the measured a* value (1.60) and chlorophyll content (1.95 mg/100 g, db) closely matched model predictions, demonstrating adequate retention of the characteristic green color of immature rice while limiting excessive browning. For nutritional quality, the experimentally measured total phenolic compound content (61.8 mg GAE/100 g, db) was in close agreement with the predicted value (62.1 mg GAE/100 g, db), indicating preservation of antioxidant potential. Likewise, the experimental expansion index (1.90) and hardness (104.3 N) were comparable to predicted values, reflecting a balanced cooked-rice texture with adequate expansion and controlled firmness. Residual diagnostic plots were employed to further evaluate model adequacy (Figure 5). The residuals versus predicted values plot showed a random scatter of residuals around zero without discernible trends, curvature, or heteroscedasticity, indicating that the assumptions of constant variance and model linearity within the response surface were reasonably satisfied. In addition, the observed versus predicted plots demonstrated a close alignment of all selected responses with the 1:1 reference line, further supporting the robustness of the selected models for optimization purposes.

Accordingly, the optimal conditions of the roasting process involved using a gas-fired unit fueled by liquefied petroleum gas (LPG) designed to generate near-infrared radiation through heating of the burner surface. The burner dimensions were 135 × 840 mm. The average fuel consumption rate was 0.68 kg h^−1^, corresponding to a heat output of 8120 kcal h^−1^ [18]. Activity-based energy analysis showed that the LPG-fired near-infrared (IR) roasting system had the lowest estimated batch-level energy demand among the roasting methods evaluated. Under optimized conditions (650 °C, 20 min, the IR-LPG system consumed 3.15 kWh per batch, equivalent to 0.227 kg LPG per batch, with an estimated carbon emission of 0.705 kg CO_2_ per batch. By comparison, conventional LPG roasting without IR heating, based on roasting conditions established in a previous immature rice study (1.0–1.5 h), was estimated to consume 9.44–14.16 kWh per batch. In contrast, a traditional firewood roasting, also based on a previous study (3 h), required 28.31 kWh per batch on the same thermal-duty basis with an estimated carbon emission of 13.43 kg biogenic CO_2_ per batch. An additional electric IR case required the same energy input (3.15 kWh per batch), while the estimated carbon emission varied depending on the electricity source, estimated to 1.21 kg CO_2_ per batch under the average Thailand grid. These results suggest that the LPG-fired IR system was the most energy-efficient option among the compared roasting processes providing lower CO_2_ emission.

Model validation has affirmed that head rice yield and a* exhibited statistically significant regression terms, acceptable R^2^ values, and strong agreement between predicted and experimental values, justifying their selection as key optimization responses. Head rice yield serves as a critical indicator of kernel structural integrity and reflects the combined effects of thermal stress, moisture redistribution, and mechanical resistance during IR roasting. Excessive or uneven heating is known to promote fissuring and kernel breakage, thereby reducing head rice yield [45]. The strong predictive performance observed in this study confirms the suitability of head rice yield as a primary indicator of processing efficiency. The a* value was retained as a representative visual quality parameter because it is highly sensitive to pigment degradation and early non-enzymatic browning reactions induced by IR heating. Changes in redness are commonly associated with chlorophyll degradation and Maillard reaction initiation, both of which strongly influence consumer perception of immature rice products [43]. Despite the statistically significant lack-of-fit detected for chlorophyll and total phenolic content (TPC) in the ANOVA, both responses were intentionally retained for optimization due to their nutritional relevance, acceptable coefficients of determination, and successful experimental validation. It is affirmed that chlorophyll content is a defining quality attribute of immature rice, contributing to its characteristic green appearance and perceived freshness. The close agreement between predicted and experimental chlorophyll values confirms that the model was sufficiently accurate for practical optimization. Similarly, the strong agreement between predicted and experimental TPC values indicates that the response surface captured the dominant trends governing phenolic behavior under the selected processing conditions.

In addition, the inclusion of cooking quality attributes—expansion index and hardness—ensured that the optimized roasting conditions translated into desirable functional performance after cooking, rather than merely improving raw grain attributes. The expansion index reflects water absorption capacity and starch gelatinization behavior. The high predictive accuracy for this response suggests that the optimized IR roasting conditions promoted controlled starch structural modification without excessive granule collapse, consistent with previous observations in thermally treated rice grains [40] with an optimal rice hardness as it is a primary determinant of cooked rice texture and consumer acceptability.

Overall, these results confirm the robustness of the developed second-order polynomial model and demonstrate that RSM-based optimization is an effective tool for controlling multiple quality attributes of infrared-roasted immature rice. The optimized IR roasting conditions achieved a balanced improvement in processing efficiency, visual quality, nutritional retention, and cooked rice performance, supporting the industrial applicability of IR roasting for high-quality immature rice production.

## 4. Conclusions

Conventional pan roasting of immature rice is energy-intensive, difficult to control, and frequently results in inconsistent quality and degradation of heat-sensitive bioactive compounds. This study demonstrated that IR roasting, coupled with response surface methodology (RSM), provides a reliable and controllable alternative for processing dough-stage glutinous rice while preserving key quality attributes. Moreover, roasting temperature (550–650 °C) and roasting time (20–40 min) significantly influenced grain yield, head rice yield, color, moisture status, chlorophyll retention, phenolic content, and cooked-rice texture. The developed quadratic models showed good predictive performance (R^2^ = 0.68–0.83), enabling effective multi-response optimization. Superimposed contour analysis identified 650 °C for 20 min as the optimal condition, achieved a favorable balance among grain integrity, green color preservation, antioxidant potential, and desirable cooked-rice texture. Experimental validation confirmed the reliability of the optimized conditions, with the measured yield, head rice yield, a* value, chlorophyll content, total phenolic compounds, expansion index, and hardness, closely matching model predictions.

In summary, this study establishes a systematic optimization framework for IR roasting of immature rice, highlighting its potential as a green-energy, scalable processing technology. IR roasting enables precise control over quality development while reducing processing severity, supporting its application in value-added cereal production. Future studies should address scale-up feasibility, energy efficiency, sensory acceptance, and varietal responses to further facilitate industrial implementation.

## Figures and Tables

**Table 1:** Physical and chemical quality attributes of infrared-roasted immature rice at different roasting temperatures (550–650 °C) and roasting times (20–40 min).

Temperature (°C)	Time (min)	Moisture Content (%)	Water Activity	Yield (%)	Head Rice Yield (%)	Color			Chlorophyll (mg/100 g Dried Sample)	Total Phenolic Compound (mg GAE/100 g Dried Sample)
						L*	a*	b*		
550	20	12.5 ± 0.5 ^ab^	0.7 ± 0.0 ^a^	30.8 ± 1.7 ^Ae^	87.7 ± 1.0 ^a^	46.5 ± 0.5 ^ab^	1.9 ± 0.2 ^c^	26.9 ± 0.8 ^f^	1.4 ± 0.3 ^bc^	66.4 ± 5.5 ^b^
550	30	12.6 ± 0.2 ^ab^	0.6 ± 0.0 ^ab^	32.6 ± 3.0 ^Ae^	88.3 ± 1.1 ^a^	47.8 ± 0.2 ^a^	1.7 ± 0.2 ^c^	27.0 ± 0.7 ^e^	1.1 ± 0.1 ^de^	59.2 ± 5.5 ^bcd^
550	40	13.3 ± 0.2 ^a^	0.6 ± 0.0 ^b^	33.1 ± 1.3 ^Af^	86.9 ± 1.1 ^a^	47.2 ± 0.7 ^ab^	1.8 ± 0.2 ^c^	27.5 ± 0.2 ^e^	1.0 ± 0.0 ^e^	56.9 ± 0.5 ^cd^
600	20	12.4 ± 0.2 ^ab^	0.6 ± 0.0 ^ab^	32.9 ± 3.1 ^Ae^	80.8 ± 0.7 ^b^	47.0 ± 0.6 ^ab^	1.8 ± 0.4 ^c^	26.1 ± 0.2 ^f^	1.6 ± 0.2 ^b^	61.2 ± 6.2 ^bcd^
600	30	13.2 ± 0.6 ^a^	0.6 ± 0.0 ^b^	33.4 ± 5.5 ^Ae^	89.8 ± 1.0 ^a^	47.2 ± 0.3 ^ab^	1.9 ± 0.1 ^c^	27.7 ± 0.7 ^e^	1.6 ± 0.9 ^bc^	55.5 ± 5.1 ^d^
600	40	9.3 ± 0.9 ^c^	0.4 ± 0.0 ^c^	25.2 ± 1.3 ^Af^	77.3 ± 2.6 ^c^	46.5 ± 0.5 ^bc^	3.6 ± 0.2 ^b^	26.9 ± 0.7 ^e^	1.4 ± 0.1 ^bc^	59.5 ± 4.2 ^bcd^
650	20	12.3 ± 0.4 ^ab^	0.6 ± 0.0 ^ab^	29.5 ± 3.0 ^Be^	88.5 ± 1.0 ^a^	47.8 ± 0.4 ^a^	1.7 ± 0.7 ^c^	26.0 ± 0.1 ^f^	2.3 ± 0.2 ^a^	64.0 ± 1.7 ^bc^
650	30	11.9 ± 0.9 ^b^	0.6 ± 0.1 ^b^	28.2 ± 0.9 ^Be^	77.5 ± 2.6 ^c^	46.5 ± 0.5 ^a^	1.9 ± 0.2 ^c^	26.9 ± 0.7 ^e^	1.3 ± 0.1 ^cd^	57.3 ± 5.8 ^cd^
650	40	5.7 ± 0.3 ^d^	0.3 ± 0.0 ^d^	21.8 ± 6.4 ^Bf^	66.4 ± 0.3 ^d^	44.3 ± 0.3 ^c^	5.8 ± 0.5 ^a^	26.9 ± 0.3 ^e^	0.9 ± 0.0 ^e^	88.5 ± 2.0 ^a^

Note: Values are mean ± standard deviation (*n* = 3). Differences were significant at *p* ≤ 0.05. ^a–d^ Mean values in the same column with different letters indicate significant differences (*p* ≤ 0.05) among treatments. ^A,B^ Mean values in the same column with different letters indicate significant differences (*p* ≤ 0.05) among roasting temperature treatments. ^e,f^ Mean values in the same column with different letters indicate significant differences (*p* ≤ 0.05) among roasting time treatments.

**Table 2:** Cooked immature rice quality obtained from different roasting temperatures and times.

Temperatures (°C)	Time (min)	Bulk Density (kg/m^3^)	Expansion Index	Texture Profile Analysis			
				Hardness (N)	Chewiness (kgf.mm)	Cohesiveness	Adhesiveness (kgf.mm) ^ns^
550	20	1.92 ± 0.02 ^cd^	243.56 ± 0.81 ^f^	79.90 ± 11.78 ^Ch^	8.44 ± 1.12 ^b^	0.17 ± 0.02 ^h^	0.05 ± 0.06
550	30	1.97 ± 0.06 ^bcd^	334.20 ± 3.93 ^b^	86.56 ± 6.62 ^Ah^	10.14 ± 2.86 ^b^	0.16 ± 0.02 ^h^	0.27 ± 0.26
550	40	1.99 ± 0.02 ^bcd^	307.39 ± 9.45 ^cd^	85.98 ± 14.44 ^Bg^	7.94 ± 0.85 ^b^	0.18 ± 0.03 ^g^	0.03 ± 0.02
600	20	2.00 ± 0.04 ^bc^	309.75 ± 7.41 ^c^	87.68 ± 4.66 ^Ch^	6.73 ± 3.52 ^b^	0.17 ± 0.04 ^h^	0.13 ± 0.10
600	30	2.01 ± 0.04 ^bc^	288.04 ± 7.88 ^e^	91.66 ± 11.55 ^Bh^	8.02 ± 1.24 ^b^	0.17 ± 0.02 ^h^	0.10 ± 0.09
600	40	2.25 ± 0.03 ^a^	310.14 ± 6.23 ^c^	113.76 ± 17.76 ^Ag^	9.19 ± 1.24 ^b^	0.17 ± 0.01 ^g^	0.17 ± 0.16
650	20	1.90 ± 0.01 ^d^	295.71 ± 6.82 ^de^	112.33 ± 9.80 ^Cg^	9.45 ± 3.04 ^b^	0.16 ± 0.03 ^h^	0.06 ± 0.05
650	30	2.04 ± 0.10 ^b^	340.14 ± 6.90 ^b^	113.30 ± 5.06 ^Bg^	7.90 ± 1.82 ^b^	0.15 ± 0.03 ^h^	0.30 ± 0.29
650	40	2.29 ± 0.07 ^a^	501.22 ± 10.75 ^a^	146.90 ± 13.03 ^Ah^	15.86 ± 4.36 ^a^	0.22 ± 0.03 ^g^	0.16 ± 0.16

Note: The data shows the average and standard deviation. ^a–f^ Mean values in the same column with different letters indicate significant differences (*p* ≤ 0.05) among 3 × 3 treatment combinations. ^A,B,C^ Mean values in the same column with different letters indicate significant differences (*p* ≤ 0.05) among roasting temperature levels, averaged over roasting time. ^g,h^ Mean values in the same column with different letters indicate significant differences (*p* ≤ 0.05) among roasting time level, averaged over roasting temperature. ^ns^ is non-significant different among treatments.

**Table 3:** Analysis of variance and coefficient of multiple regression model for several rice quality responses.

Source	DF	Roasted Immature Rice					Cooked Immature Rice			
		Yield (%)	Head Rice Yield (%)	a*	Chlorophyll (mg/100 g Dried Sample)	Phenolic Compound (mg GAE/100 g Dried Sample)	Expansion Index	Hardness (N)	Cohesiveness	Adhesiveness (kgf.mm)
**Analysis of variances**										
Regression	5	350.47 *	1244.51 *	44.15 *	3.54 *	2055.5 *	0.49 *	10,635.80 *	0.008 *	0.16 ns
Linear	2	227.68 *	815.54 *	25.64 *	2.45 *	459.8 *	0.32 *	9455.10 *	0.003 ns	0.04 ns
Square	2	42.35 ns	92.10 *	5.05 *	0.32 *	727 *	0.04 *	557.10 ns	0.003 *	0.13 ns
Interaction	1	80.45 *	336.87 *	13.48 *	0.77 *	868.7 *	0.08 *	623.50 *	0.001 ns	0.00 ns
Residual Error	21	247.70	236.19	7.08	0.98	729.6	0.07	2755.60	0.013	0.53
Lack-of-fit	3	**36.27 ns**	200.86 *	2.84 *	0.61 *	400.7 *	**0.02 ns**	**449.80 ns**	**0.002 ns**	**0.05 ns**
Total	26	598.17	1507.70	51.22	4.52	2785.1	0.52	13,391.40	0.02	0.70
R^2^ (%)		48.70	**78.40**	**82.90**	**73.10**	**67.60**	**83.30**	**74.50**	23.00	4.60
**Coefficient of multiple regression analysis**										
$\beta_{0}$		32.10 *	85.24 *	1.84 *	1.45 *	52.87 *	2.05 *	92.90 *	0.15 *	0.18 *
$\beta_{1}$		−2.75 *	−5.09 *	0.68 *	0.16 *	4.51 *	0.058 *	20.07 *	0.002 ns	0.048 ns
$\beta_{2}$		−2.26 *	−4.40 *	0.98 *	−0.33 *	2.22 ns	0.12 *	11.07 *	0.013 *	−0.016 ns
$\beta_{11}$		−1.08 ns	−0.07 ns	0.005 ns	−0.19 *	6.63 *	−0.070 *	6.41 ns	0.004 ns	0.067 ns
$\beta_{22}$		−2.43 *	−3.92 *	0.92 *	0.13 ns	8.79 *	0.051 *	7.20 ns	0.024 *	−0.12 ns
$\beta_{12}$		−2.59 *	−5.29 *	1.06 *	−0.25 *	0.51 *	0.083 *	7.21 *	0.011 ns	0.003 ns

Note: Regression equation: $y_{n}=\beta_{0}+\beta_{1}x_{1}+\beta_{2}x_{2}+\beta_{11}x_{1}^{2}+\beta_{22}x_{2}^{2}+\beta_{12}x_{1}x_{2}$. $x_{1}$: roasting temperature (°C); $x_{2}$: roasting time (min); *: different letters indicate significant differences (*p* ≤ 0.05); ns: not different letters indicate significant differences (*p* ≤ 0.05).

**Table 4:** Acceptable ranges of response variables used for predicting optimal infrared roasting conditions of immature rice.

Response Variable	Equation in Terms of Coded Factors	(R^2^)	Lower Limit	Upper Limit
Head rice yield (%)	Y_1_ = 85.23 − 5.09x_1_ − 4.40x_2_ − 0.07x_1_^2^ − 3.91x_2_^2^ − 5.29x_1_x_2_	0.78	60.0	100.0
a* value	Y_2_ = 1.84 + 0.69x_1_ + 0.98x_2_ + 0.005x_1_^2^ + 0.91x_2_^2^ + 1.06x_1_x_2_	0.83	−3.10	2.20
Chlorophyll	Y_3_ = 1.45 + 0.16x_1_ − 0.33x_2_ − 0.19x_1_^2^ + 0.13x_2_^2^ − 0.25x_1_x_2_	0.73	0.70	5.80
Total phenolic compound	Y_4_ = 52.87 + 4.54x_1_ + 2.22x_2_ + 6.63x_1_^2^ + 8.79x_2_^2^ + 8.50x_1_x_2_	0.68	62.0	120.0
Expansion index	Y_5_ = 2.05 + 0.05x_1_ + 0.12x_2_ − 0.06x_1_^2^ + 0.05x_2_^2^ + 0.08x_1_x_2_	0.83	0.90	2.20
Hardness (N)	Y_6_ = 92.90 + 20.07x_1_ + 11.06x_2_ + 6.40x_1_^2^ + 7.20x_2_^2^ + 7.20x_1_x_2_	0.75	80.0	112.0

Note: The acceptable ranges were established based on quality standards, relevant literature, and preliminary experimental observations, and were used as constraints for multi-response optimization using response surface methodology.

**Table 5:** Model validation at optimum infrared roasting conditions (650 °C, 20 min) of immature rice.

Response	Predicted Value	Experimental Values ^ns^
Head rice yield (%)	85.9	88.2 ± 1.5
a* value	1.40	1.60 ± 0.1
Chlorophyll content (mg/100 g dried sample)	2.20	1.95 ± 0.0
Phenolic compound (mg GAE/100 g dried sample)	62.1	61.83 ± 0.8
Expansion index	1.90	1.85 ± 0.1
Hardness (N)	108.0	104.33 ± 3.3

Note: ns = no significant difference in the same row (*p* ≤ 0.05).

## Data Availability

The raw data supporting the conclusions of this article will be made available by the authors on request.
